# Supplementary material for: Diversity of meso-scale architecture in human and non-human connectomes
Source: Nat Commun. 2018 Jan 24;9:346. doi: 10.1038/s41467-017-02681-z (PMC5783945; doi:10.1038/s41467-017-02681-z)
Supplement: Supplementary file 1 — Supplementary Information [file 41467_2017_2681_MOESM1_ESM.pdf]

Supplement to: Diversity of meso-scale architecture in human and non-human connectomes

Richard F. Betzel[1], John D. Medaglia[2], and Danielle S. Bassett[1,3]

[1]Department of Bioengineering
[2]Department of Psychology
[3]Department of Electrical and Systems Engineering
University of Pennsylvania, Philadelphia, PA, 19104

December 16, 2017

Supplementary Methods

In this supplement we describe four additional inter-regional, non-human connectome datasets which we analyzed using the same methods and techniques as the human data. Whereas the human connectome data described in the main text were reconstructed *in vivo* from diffusion weighted images using deterministic tractography algorithms, the procedures used to reconstruct the non-human data were more varied, ranging from retrograde tract tracing to meta-analysis. These data allow us to assess whether the differences we observed between the WSBM and Q_{max} techniques are a consequence of the procedures used to reconstruct the connectome data. We also report a similar set of analyses carried on the *C. elegans* chemical synapse network. Unlike the region-level networks described in the main text and earlier in this supplement, the analysis of *C. elegans* allows us to probe the meso-scale structure of a complete neural system at the cellular level. In general, the analyses we present in this supplement corroborate those in the main text. We also report several confirmatory and robustness analyses. These include showing that the WSBM algorithm consistently converges to similar solutions, a comparison of communities obtained using empirical and randomly rewired networks, and a demonstration that our reported results do not depend trivially on the partition of the human brain in parcels (network nodes).

Supplementary Note 1: Non-human connectome data

Mouse

In addition to human data, we analyzed a mouse connectome reconstructed from tract-tracing experiments made publicly available by the Allen Brain Institute [3]. Tracers were tracked from a series of injection sites to ispi- and contra-lateral brain regions. We focus on a reprocessed version of these data in which the mouse brain was parcellated into $N = 112$ regions (56 per hemisphere) and edge weights defined as volume-normalized number of connections between regions [4]. Rather than use the whole-brain connectome described in that report (generated by copying and mirroring tract-tracing data from one hemisphere to the other), we analyzed a single cerebral hemisphere ($N = 56$ regions) for which complete connectivity data was available. Due the directed nature of the tract-tracing experiments, the resulting network was asymmetric (i.e. $A_{ij} \neq A_{ji}$) (Supplementary Figure 1A).

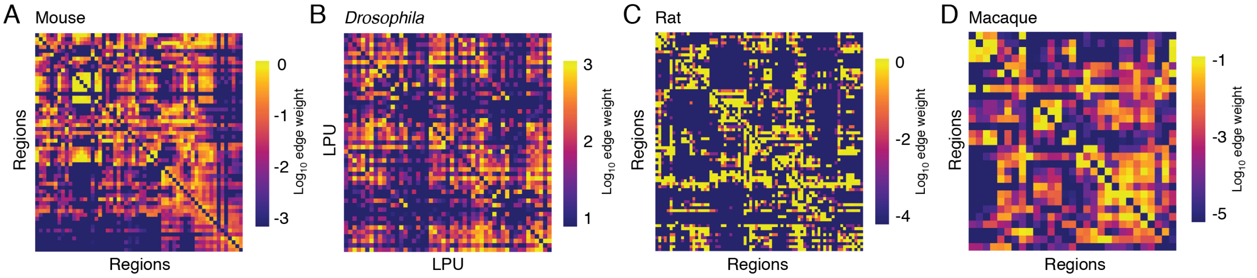

Supplementary Figure 1: **Non-human connectome data.** Connectivity matrices for the four non-human connectome datasets.

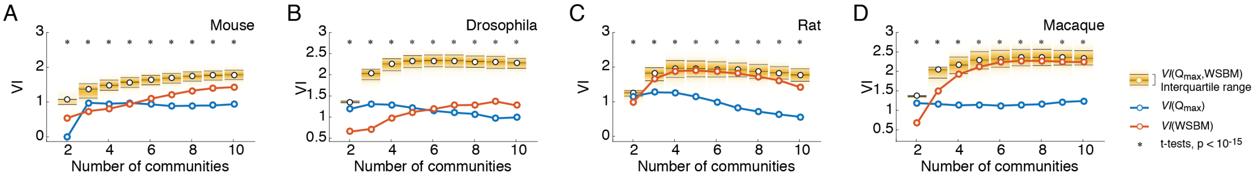

Supplementary Figure 2: **Partition dissimilarity for non-human connectome data.** We show mean within-technique partition dissimilarity (variation of information; VI) in blue and orange for Q_{max} and the WSBM, respectively, along with the between-technique dissimilarity (yellow). Panels (A-D) depict VI as a function of the number of communities for mouse, *Drosophila*, rat, and macaque, respectively.

Drosophila

We also analyzed a network reconstructed from 12,995 projection neurons in the female *Drosophila* brain [5,6]. Neurons were aggregated among $N = 49$ local processing units (LPUs), which represent network nodes. The resulting network is directed and weighted and has been reported elsewhere [6,7] (Supplementary Figure 1B).

Rat

We also analyzed a rat cortical network [8]. This network was constructed by collating reports on rat tract-tracing experiments [9], extracting information from those reports regarding the existence of connections (resulting in >16000 connections), and based on the consistency and quality of those results, assigning a single weight to existing inter-regional connections. The result is a directed network of $N = 73$ cortical regions spanning a single hemisphere (Supplementary Figure 1C).

Macaque

We also analyzed an incomplete macaque connectome documenting the connections among $N = 29$ cortical regions [10,11]. As with some of the other datasets, the macaque connections were established from retrograde tract-tracing experiments and were weighted as fraction of labeled neurons ("the number of labeled neurons in a given source area relative to the total number of labeled neurons in the brain for any given injection"). The result is a weighted and directed connectivity matrix (Supplementary Figure 1D).

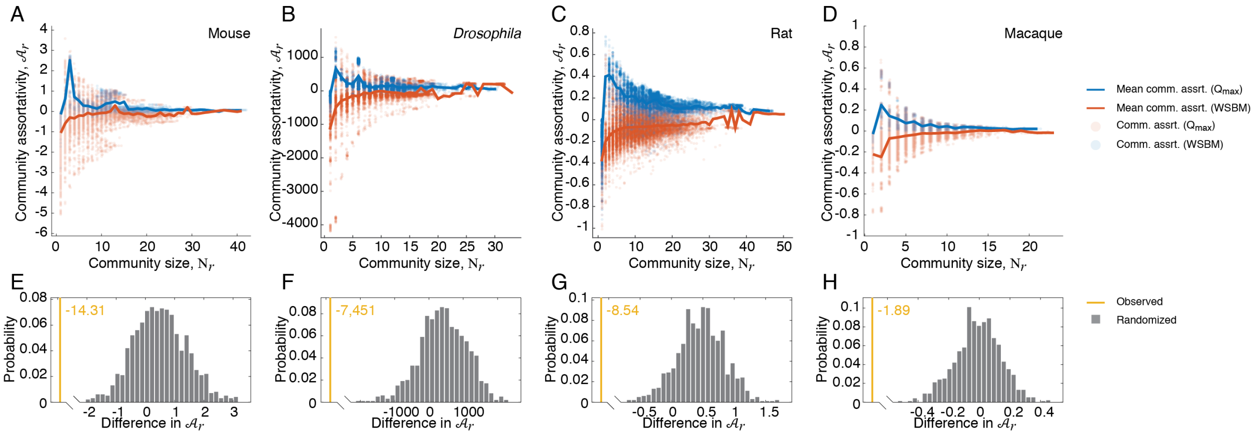

Supplementary Figure 3: **Community assortativity for non-human connectome data.** We show the assortativity of each detected community for both the WSBM (orange) and Q_{max} (blue) as a function of community size. The solid curves represent the mean community assortativity for each technique as a function of community size. Panels (*A*-*D*) depict scores for mouse, *Drosophila*, rat, and macaque, respectively. The accompanying panels (*E* - *H*) are the results of the functional data analysis, in which we compared the mean community assortativity curves. Yellow lines represent the observed test statistic; gray bars represent the null distribution.

Additional data

As with the human dataset, each non-human dataset was accompanied by system labels. For the mouse data, we used the high-resolution parcellation of the mouse brain into eight systems described in [4] (three subdivisions of an olfactory/hippocampal network, a somatomotor/sensory/motor system, a brainstem/cerebellar system, and three subdivisions of an audition/visual network). For *Drosophila* we used a division of the network into five systems (audition/mechanosensation, olfactory, premotor, visual-left, visual-right) [6]. For rat we used a division into three systems (sensory/motor, poly-association, and cortical subplate) [8]. For macaque we used a division into six systems [12] (prefrontal, parietal, temporal, occipital, frontal, and other).

Finally, the mouse dataset included gene expression profiles for each region. These data were originally made available by the Allen Brain Institute and were obtained using high-throughput *in situ* hybridization and acquization, and included 3,380 of the $>$ 20,000 genes assayed that survived quality control. See [4] for criteria for inclusion of this particular set of genes. From these gene expression data, we computed the correlation of each pair of regions' expression profiles, resulting in a square matrix.

Comparing the WSBM with Q_{max}

As in the main text, we compared the partitions detected using the WSBM with those detected using Q_{max}. This involved a partition-level comparison using variation of information (VI), a community-level comparison using the community assortativity measure, $\mathcal{A}_r$, a regional-level analysis based on a regional assortativity score, a_i, and functional data analysis.

For the partition-level analyses, we observed that the partitions detected using either the WSBM or Q_{max} were more internally consistent than they were similar to one another as we varied the number of communities from $K = 2$ to $K = 10$ (Supplementary Figure 2A-E) (t-tests, $p < 10^{-15}$). We found similarly consistent results when we examined community assortativity (Supplementary Figure 3A-H), with Q_{max} communities being more assortative than WSBM communities (controlling for size, $p < 10^{-3}$), and when we

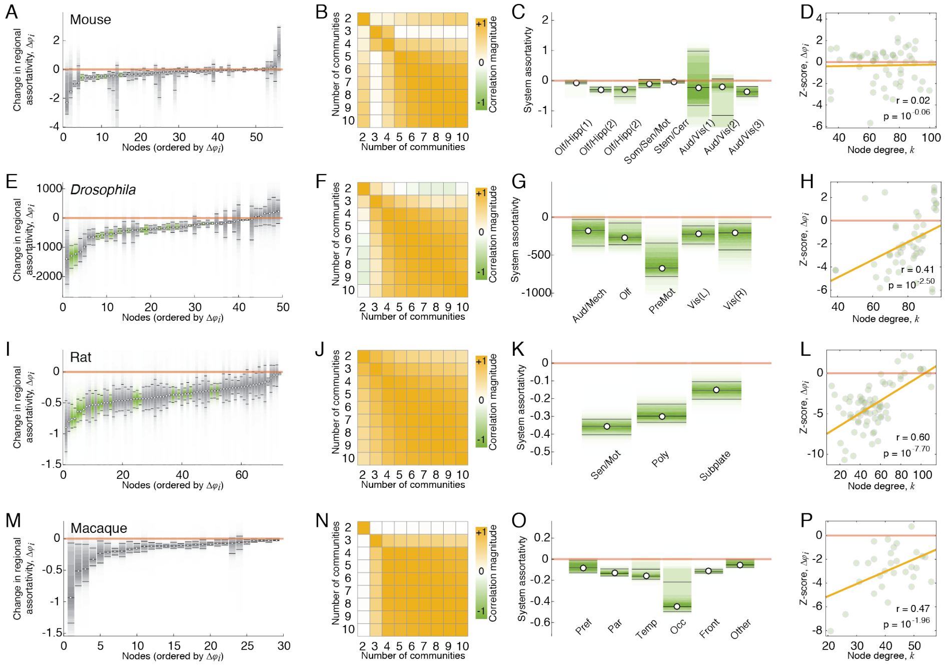

Supplementary Figure 4: **Regional assortativity for non-human connectome data.** Panels (A,E,I,M) depict differences in regional assortativity scores, $\Delta\phi_i$, for mouse, *Drosphila*, rat, and macaque. Panels (B,F,J,N) show the similarity (correlation) of changes in regional assortativity as a function of the number of communities. Panels (C,G,K,O) show the same regional assortativity changes grouped according to functional systems for each connectome dataset. Panels (D,H,L,P) show the relationship of regional assortativity to node degree.

examined regional assortativity (Supplementary Figure 4), with the majority of regions exhibiting decrements in regional assortativity under the WSBM.

While these results were, overall, consistent with those reported in the main text, there were nonetheless some differences. For example, in the mouse dataset the relationship of node degree with change in regional assortativity was practically non-existent. The source of this variation is unclear, though it is important to note that, while the non-human datasets are reconstructed using what are arguably higher-fidelity techniques, e.g. tract tracing, they nonetheless suffer from peculiarities, notably incompleteness. The macaque connectome includes connection data on only 29 of 91 brain areas while the mouse and rat data include only a single hemisphere. For this reason, it is difficult to ascertain whether differences in connectome mesoscale structure across species arises due to genuine architectural differences or whether complete connectivity information would improve the consistency of results.

Maximally assortative set

In the main text, we demonstrated that there was a subset of around 75% of all network nodes whose communities were all mutually assortative – i.e. the minimum within-community connection density of all communities exceeded the maximum between-community density of any pair of communities. Here, we reproduce this analysis for the non-human connectome data. In general, these results corroborate those of the main text. In particular, we find that the maximally assortative set is never empty (Supplementary Figure 5A, E, I, M), though its size varies considerably across networks, with an average size of 0.37 ± 0.11 in *Drosophila* up to 0.58 ± 0.09 in mouse.

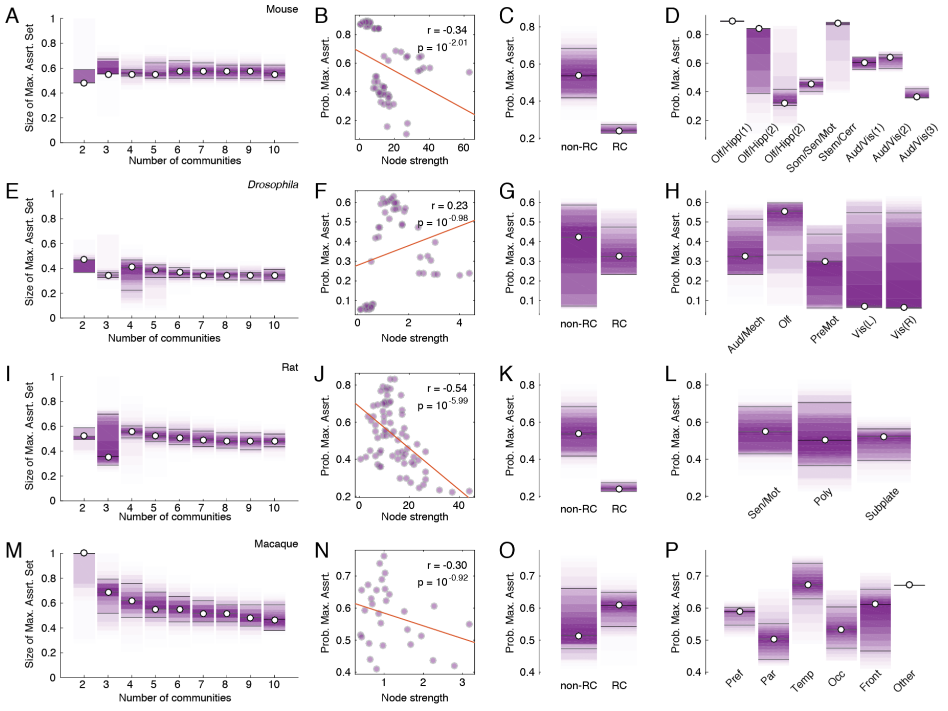

Supplementary Figure 5: **Maximaly assortative set for non-human connectome data.** We show the size of the maximally assortative set (as a fraction of total number of nodes) for the (*A*) mouse, (*E*) *Drosophila*, (*I*) Rat, and (*M*) macaque connectomes. For each connectome dataset, we also plot the probability that a node gets assigned to the maximally assortative set as a function of the logarithm of node strength (*B,F,J,N*), whether or not (*C,G,K,O*), and according to the functional system to which each node was assigned (*D,H,L,P*). The labels RC and non-RC used in panels *C,G,K,O* indicate nodes that were assigned to or not assigned to putative rich clubs See **Rich club estimation** for more details.

We also observed that, in general, participation in the maximally assortative set was negatively correlated with node strength (though we observe an opposite trend in the *Drosophila* dataset; Supplementary Figure 5F). Similarly, in terms of rich club participation in the maximally assortative set, we observed that rich club nodes were less likely to participate in the maximally assortative set than non-rich club nodes (though again, we see an opposite effect in the macaque dataset). For completeness, we also show participation in the maximally assortative set by functional system.

Community morphospace analysis

As with the human connectome data presented in the main text, we compared the morphospaces populated by communities detected using the WSBM and Q_{max} (Supplementary Figure 6). We repeated this analysis separately for each non-human connectome dataset. Unlike the human connectome data, which was directed (and so the following equality holds: $\omega_{rs} = \omega_{sr}$), the directed non-human networks allowed us to examine a greater number of community interactions. Nonetheless, the results of these analyses were entirely consistent with those described in the main text using the human connectome data. Namely, we observed that for all non-human data and over the range $K = 2, \ldots, 10$, Q_{max} discovers almost exclusively assortative community motifs. The WSBM, on the other hand, detects disproportionately more disassortative and core-periphery motifs than Q_{max}. As before, we confirm these results using functional data analysis, demonstrating the

difference in motif proportions were unexpected under a permutation-based null model (mouse, $p = 0.003$; *Drosophila*, $p < 10^{-3}$; rat, $p < 10^{-3}$; macaque, $p < 10^{-3}$).

Based on the results of the community morphospace analysis, we were able to assign individual network nodes to the motif class that they expressed differentially, assign them to bins based on their node strength, and compute the entropy of the motif class distribution within each bin (Supplementary Figure 7). As in the main text, we observed that entropy peaked for middle-strength nodes, while the lowest- and highest-strength nodes were associated with low entropies, suggesting that they participate in a much narrower range of classes. Also, as described in the main text, low-strength bins were dominated by assortative and periphery motifs while high-strength bins were dominated by core motifs.

These supplementary results, in addition to those described in the previous section, bolster the findings from the main text, and suggest that our results cannot be explained by shortcomings of any one connectome reconstruction technique. As before, these supplementary results demonstrate that connectome data can be described with a rich meso-scale architecture that is detectable with the WSBM.

Functional relevance of WSBM communities for mouse connectome

With the human data, we were able to demonstrate the functional relevance of the WSBM communities by comparing the within- and between-community densities of functional connections, which were calculated given a group-representative resting-state FC matrix. Unfortunately, we do not have comparable functional recordings for any of the non-human datasets. The mouse dataset, however, does include regional gene expression profiles and the similarity of two regions' profiles is often taken to indicate the extent to which those regions participate in a shared set of functions [13, 14].

With this assumption in mind, we generate a region-by-region matrix of gene expression correlation coefficients, which we can view as analogous to the human FC matrix. We can then impose partitions detected using the WSBM and Q_{max} onto this matrix and compute the mean within- and between-community correlation. As before, the larger the difference in these two variables, the better job a partition does in uncovering functionally meaningful communities. We computed the difference for all partitions detected from $K = 2$ to $K = 10$. We observed that over the sub-range $K = 2$ to $K = 8$, the WSBM partitions resulted in greater differences between within- and between-community correlations (one-tailed t-test; $p < 10^{-5}$). This suggests that over that same range, the WSBM detects communities of brain regions that are correlated with one another but, in general, anti-correlated between communities. Moreover, the WSBM performs better than Q_{max} (Supplementary Figure 8), indicating that on average the WSBM detects communities that are better aligned with the correlated expression profiles of brain regions in the mouse than the communities detected using Q_{max}.

Supplementary Note 2: WSBMs at the cellular level

The human connectome data analyzed in the main text and the non-human connectomes analyzed in this supplement are examples of inter-regional networks. Individual cells and populations have been aggregated into spatially-contiguous, macroscopic parcels or regions. While this approach is common and serves to reduce the dimensionality of a network (making it more manageable for analysis), it also averages over the properties of those cells and populations. If regions are homogeneous in terms of the cells they contain, then region-level analysis loses very little information. However, if a region's constituent cells exhibit heterogeneity in terms of their connectivity patterns, then we lose access to this information. It is unclear, then, how the WSBM would behave when applied to a cellular-level network.

In this section we apply the WSBM to the network of the nematode *C. elegans*. We analyze the directed and weighted network of chemical synapses described in [15]. This network consists of 279 neurons, 2194 connections, and 6394 synapses (neurons can synapse onto one another more than once). Our aim is to show that, even at this cellular scale, the WSBM identifies high levels of non-assortativity while Q_{max} does not. As we note in the main text, this does not demonstrate conclusively that the "true" meso-scale structure of *C. elegans* is composed of non-assortative communities. Instead, it complements other recent papers [16] highlighting the apparent utility of blockmodels for identifying non-trivial communities in cellular-level data.

Our analysis consisted of two components. First, using identical methods as in the main text, we constructed a morphospace of community interactions (Supplementary Figure 9). This figure demonstrates that when we use Q_{max} to uncover communities and vary the number of communities from $K = 2$ to $K = 10$, communities are *always* arranged in assortative motifs. Using the WSBM, on the other hand, we consistently identify both core-periphery and disassortative motifs.

As a second comparison of Q_{max} and the WSBM applied to *C. elegans* connectome data, we identified representative partitions for each technique as we varied the number of communities from $K = 2$ to $K = 10$. We then reordered and blocked the *C. elegans* connectivity matrix according to the communities uncovered by the WSBM (Supplementary Figure 10A). In the margins of each plot, we color-coded each node's corresponding community label as detected using Q_{max}. Had the two techniques generated similar community partitions, then we would expect the Q_{max} labels to be homogeneous within each block. However, Q_{max} community labels are heterogonously distributed within WSBM communities, demonstrating qualitatively that the two techniques uncover communities of different character.

In addition, for the representative WSBM communities, we also show the density (average weight of all possible connections) of each block (Supplementary Figure 10B). While certain pairs of communities are configured in assortative relationships, many are not. These results suggest that the WSBM identifies non-assortative communities in the *C. elegans* connectome. Seeing this structure at the cellular scale in a fully-mapped connectome further supports the conclusions of our manuscript.

Supplementary Note 3: Convergence of the WSBM across multiple repetitions

Here we summarize additional analyses of the human connectome dataset to characterize the variance of solutions obtained using the WSBM. First, we demonstrate qualitatively and visually that partitions detected using the WSBM are similar to one another. Second, we use statistical methods to quantify the observed level of similarity and show that it is much greater than what would be expected under a permutation-based null model. These results demonstrate that the WSBM partitions are statistically reliable, supporting the use of the WSBM for community detection in network neuroscience.

In the main text we used the WSBM to partition brain networks into $K = 2, \ldots, 10$ communities. Because the WSBM algorithm was non-deterministic, we ran it multiple times from different intimal conditions (250 repetitions), generating partition ensembles at each value of K. Here, we provide visual evidence suggesting that the partitions comprising each ensemble are, in fact, similar to one another. Specifically, we compute for each partition ensemble its association matrix, $\mathbf{T} \in \mathbb{R}^{N \times N}$, whose element, T_{ij}, is equal to the fraction of partitions in which nodes, i and j, are assigned to the same community. If partitions were dissimilar to one another, the association matrix would exhibit no structure. In Figure 11 we show examples of association matrices generated from partitions obtained using the WSBM. Note that these matrices exhibit structure in the form of non-uniform community co-assignment, providing visual confirmation that partitions generated by the WSBM are relatively consistent across multiple runs of the algorithm.

Next, we quantify the average similarity of partitions to one another and show that this level of similarity is much greater than what is expected by chance. As in the main text, we use variation of information (VI) to quantify the similarity of two partitions to one another. We define the average similarity of each partition ensemble as the mean pairwise VI across all possible pairs of partitions. To show that the detected partitions are more similar to one another than expected by chance, we compare the observed mean pairwise VI of each partition ensemble against a null distribution generated by a permutation-based null model in which a node's community assignment is swapped with that of another in the same partition with probability r. We vary the value of r from $r \approx 0.0017$ to $r = 1$ in 30 logarithmically-spaced steps and generate 100 null values at each step. We then perform one-tailed non-parametric t-tests that the observed mean pairwise VI is less than that of the null distribution and find that even when r is small, the observed value is statistically smaller than expected by chance ($p < 10^{-2}$; corrected for multiple comparisons with a false-discovery rate of 0.05) (Supplementary Figure 12). These results provide statistical evidence that the partitions detected using the WSBM are, in fact, more similar to one another than expected. These results extend and complement the visual evidence presented earlier.

Finally, it is worth noting that there are some reasons that we might expect partitions detected using the WSBM to be more variable than those detected using Q_{max}. Both algorithms are tasked with estimating nodes' community assignments. This problem is, of course, computationally intractable for all but the most trivial cases [21]. However, the problem is compounded for the WSBM, which must also estimate for every pair of communities a binary connection probability and the mean/variance of edges that fall between those communities. All else being equal, this means that the space of possible solutions is much larger for the WSBM than Q_{max}, leading to many near-optimal solutions on repeated runs.

Supplementary Note 4: Application of the WSBM to rewired networks

The WSBM is a flexible tool for detecting communities in networks using statistical inference. To properly contextualize the results presented in the main text, we applied the blockmodel to randomly-rewired networks. Specifically, we compared the observed brain network to networks with precisely the same degree sequence and approximately the same strength sequence. This process entailed first using a standard edge rewiring algorithm to rewire the observed network while preserving its exact degree sequence [1]. However, this procedure does not preserve nodes' strengths. To approximate the observed strength sequence, we randomly swapped the weights of existing edges and, using a simulated annealing algorithm, found configurations of edge weights such that nodes' strengths were minimally different from that of the observed network (Supplementary Figure 13A). We repeated the algorithm 100 times, generating 100 realizations of the rewired network (Supplementary Figure 13B).

We then used the WSBM to uncover the mesoscale structure of each rewired network. We varied the number of communities from $K = 2$ to $K = 10$ and repeated the algorithm 10 times. Next, we calculated the assortativity of each detected community based on the connection pattern of the rewired network. We found that in randomly rewired networks, the assortativity of communities detected using the WSBM was far less than that of the observed network. This is because the rewiring procedure tends to reduce the number of triangles and cliques in the network. Because these structures reinforce assortative communities, a reduction in their prevalence corresponds to a reduction in the overall assortativity of communities (Supplementary Figure 14). We traced out the average assortativity of communities as a function of community size and compared the resulting curves using functional data analysis. We found that the assortativity of communities in the observed network was significantly greater than that of the communities detected in the rewired networks ($p < 10^{-4}$).

Next, we submit the rewired networks to a morphospace analysis. As in the main text, this process entailed enumerating and classifying all two-community interaction motifs as "assortative", "core-periphery", or "disassortative" (Supplementary Figure 15A). This process was repeated as we varied the number of communities from $K = 2$ to $K = 10$. For each k, we calculated the proportion of motifs within each class (Supplementary Figure 15B-D). From this analysis we found that the rewired networks resulted in a decrease in the fraction of assortative motifs. In parallel, this reduction in assortative motifs was accompanied by an increase in core-periphery and disassortative motifs.

These additional analyses demonstrate that observed brain networks exhibit different community statistics compared to rewired brain networks. These findings inform the results in the main text. The application of the WSBM to brain networks results in less assortative communities than if Q_{max} had been used to detect communities. This level of disassortativity, however, is not as severe as in random networks, suggesting that the observed brain networks, in fact, maintain an unexpected level of assortative communities. This is an important point, as the functional and evolutionary advantages of assortative community structure have been well-documented [2], indicating that brains may balance these advantages with whatever additional advantages are conferred by possessing a small proportion of non-assortative communities.

Supplementary Note 5: Robustness of findings to variation in node definition

In the main text, we studied human connectome data in which we defined network nodes to be $N = 128$ cortical and sub-cortical regions of interested based on a sub-division of a popular brain atlas [17,18]. Based on analysis of networks defined in this way, we made claims about a regional diversity index, stating that

(1) it is peaked in subcortical and control systems, and (2) that individual variation in diversity index is predictive of subjects' accuracy on Stroop and Navon tasks, which are thought to tax control systems.

In general, a brain network's organization and statistical properties will depend upon how its nodes are defined [19]. To avoid the possibility that the results we presented in support of the above claims are biased by our choice of node definition, we repeat our analyses using alternative atlases to define the network nodes. Specifically, we compare the results in the main text with those obtained at different "scales": one coarser ($N = 82$ nodes) and one finer ($N = 233$ nodes) sub-division of the Desikan-Killiany atlas [18].

Our approach for analyzing these additional data was exactly the same as what we reported in the main text. We generated group-representative networks and used the WSBM to partition each them into $K = 2$ to $K = 10$ communities. From these communities, we defined community motifs and calculated region-level diversity indices. A direct comparison of these indices across scales is impossible because of the different number of nodes. Instead, we computed system-level diversity scores by averaging the diversity of all brain regions assigned to a given cognitive system [20]. Thus, for each network scale we obtained eight scores that summarized the mean diversity of each system. We compared these scores with those reported in the main text using a Spearman correlation. We found that, though imperfect, the correlations were positive ($r = 0.64$ and $r = 0.33$ for the 82-node and 233-node networks). We note that due to the small number of observations, these correlations are not statistically significant ($p = 0.09$ and $p = 0.43$). Nonetheless, the positive relationship indicates shared variance and a broad (yet imperfect) correspondence across scales.

Next, we sought to assess whether inter-subject variation in regional diversity indices was correlated with performance on the cognitive task. To this end, we used the WSBM to uncover communities in single-subject connectomes, calculated community motifs and diversity indices, and computed the correlation of regional diversity indices with combined accuracy on Stroop and Navon tasks (after regressing out total network weight). As before, to facilitate comparison across the different-sized networks we calculated the mean correlation of nodes assigned to each cognitive system [20]. We varied the number of communities from $K = 2$ to $K = 10$ and calculated the correlation of all system-level scores, obtaining coefficients of $r = 0.32$ and $r = 32$ ($p < 0.01$) for the 82-node and 233-node networks, respectively (Supplementary Figure 17A, B). As with the previous section, the comparison between scales was not perfect, but confirmed similar overall patterns, suggesting that our results were robust to reasonable variation in choice of parcellation.

References

[1] Maslov, S. & and Sneppen, K. Specificity and stability in topology of protein networks. *Science*, **296**, 910–913, 2002.

[2] Sporns, O. & Betzel, R.F. Modular brain networks. *Annu. Rev. Psychol.*, **67**, 613-640, (2016).

[3] Oh, S.W. *et al.* A mesoscale connectome of the mouse brain. *Nature*, **508**, 207–214, 2014.

[4] Rubinov, M. *et al.* Wiring cost and topological participation of the mouse brain connectome. *Proc. Natl. Acad. Sci. U.S.A.*, **112**, 10032–10037, 2015.

[5] Chiang, A.S. *et al.* Three-dimensional reconstruction of brain-wide wiring networks in drosophila at single-cell resolution. *Curr. Biol.*, **21**, 1–11, 2011.

[6] Shih, C.T. *et al.* Connectomics-based analysis of information flow in the drosophila brain. *Curr. Biol.*, **25**, 1249–1258, 2015.

[7] Worrell, J.C. *et al.* Signal spreading and sensory-motor integration in the drosophila connectome. *Netw. Neurosci*, 1–28, 2017.

[8] Bota, M., Sporns, O. & Swanson, L.W. Architecture of the cerebral cortical association connectome underlying cognition. *Proc. Natl. Acad. Sci. U.S.A.*, **112**, E2093–E2101, 2015.

[9] Bota, M., Dong, H.W. & Swanson, L.W. Brain architecture management system. *Neuroinformatics*, **3**, 15–47, 2005.

[10] Markov, N.T. *et al.* A weighted and directed interareal connectivity matrix for macaque cerebral cortex. *Cereb. Cortex*, **24**, 17-36, 2012.

[11] Markov, N.T. *et al.* The role of long-range connections on the specificity of the macaque interareal cortical network. *Proc. Natl. Acad. Sci. U.S.A.*, **110**, 5187–5192, 2013.

[12] Markov, N.T. *et al.* Cortical high-density counterstream architectures. *Science*, **342**, 1238406, 2013.

[13] Lamb, J. *et al.* The connectivity map: using gene-expression signatures to connect small molecules, genes, and disease. *Science*, **313**, 1929–1935, 2006.

[14] Subramanian, A. *et al.* Gene set enrichment analysis: a knowledge-based approach for interpreting genome-wide expression profiles. *Proc. Natl. Acad. Sci. U.S.A.*, **102**, 15545–15550, 2005.

[15] Varshney, L.R. *et al.* Structural properties of the caenorhabditis elegans neuronal network. *PLoS Comput. Biol.*, **7**, e1001066, 2011.

[16] Pavlovic, D.M. *et al.* Stochastic blockmodeling of the modules and core of the caenorhabditis elegans connectome. *PloS One*, **9**, e97584, 2014.

[17] Cammoun, L. *et al.* Mapping the human connectome at multiple scales with diffusion spectrum mri. *J. Neurosci. Methods*, **203**, 386–397, 2012.

[18] Desikan, R.S. *et al.* An automated labeling system for subdividing the human cerebral cortex on mri scans into gyral based regions of interest. *Neuroimage*, **31**, 968–980, 2006.

[19] Zalesky, A. *et al.* Whole-brain anatomical networks: does the choice of nodes matter? *Neuroimage*, **50**, 970–983, 2010.

[20] Yeo, B.T.T. *et al.* The organization of the human cerebral cortex estimated by intrinsic functional connectivity. *J. Neurophysiol.*, **106**, 1125–1165, 2011.

[21] Fortunato, S. Community detection in graphs. *Phys. Rep.*, **486**, 75–174, 2010.

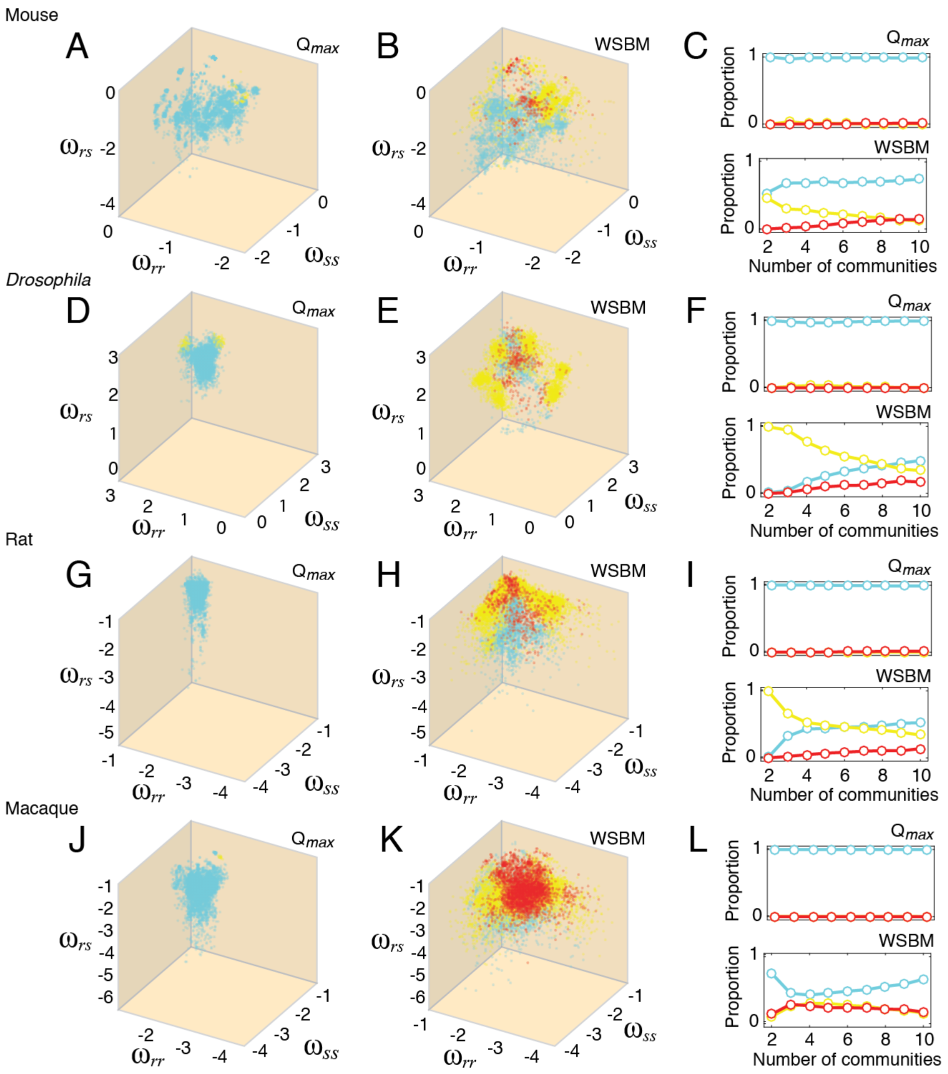

Supplementary Figure 6: **Community morphospace analysis of non-human connectome data.** Rows 1-4 depict mouse, *Drosophila*, rat, and macaque data. Panels (*A*,*D*,*G*,*J*) Community morphospaces constructed based on Q_{max} partitions. Panels (*B*,*E*,*H*,*K*) Community morphospaces constructed based on WSBM partitions. Panels (*C*,*F*,*I*,*L*) Motif proportions for both Q_{max} (*top*) and the WSBM (*bottom*) as a function of the number of detected communities.

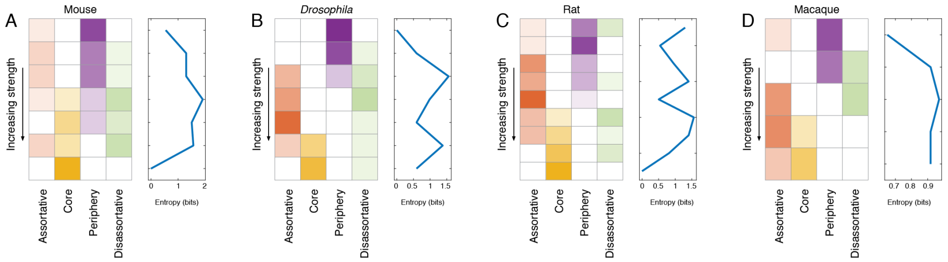

Supplementary Figure 7: **Relative proportion of community motifs as a function of node strength.** (*A*) Mouse, (*B*) *Drosophila*, (*C*) rat, and (*D*) macaque. For each connectome dataset, we show the relative proportion of each motif type (*left*) and the entropy within each bin (*right*).

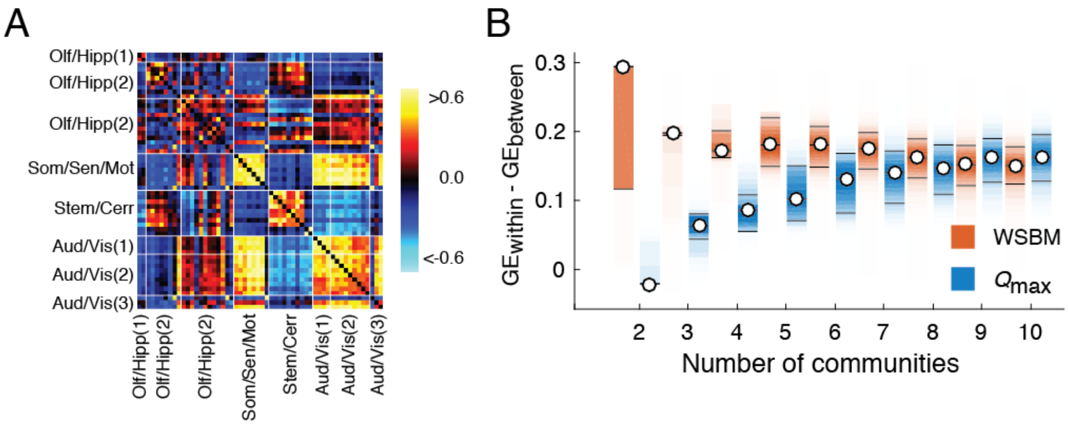

Supplementary Figure 8: **Correlated gene expression profiles and the WSBM in mouse data.** (*A*) Correlation matrix of gene expression profiles. (*B*). Difference in within- *versus* between-community correlations for both the WSBM (orange) and Q_{max} (blue).

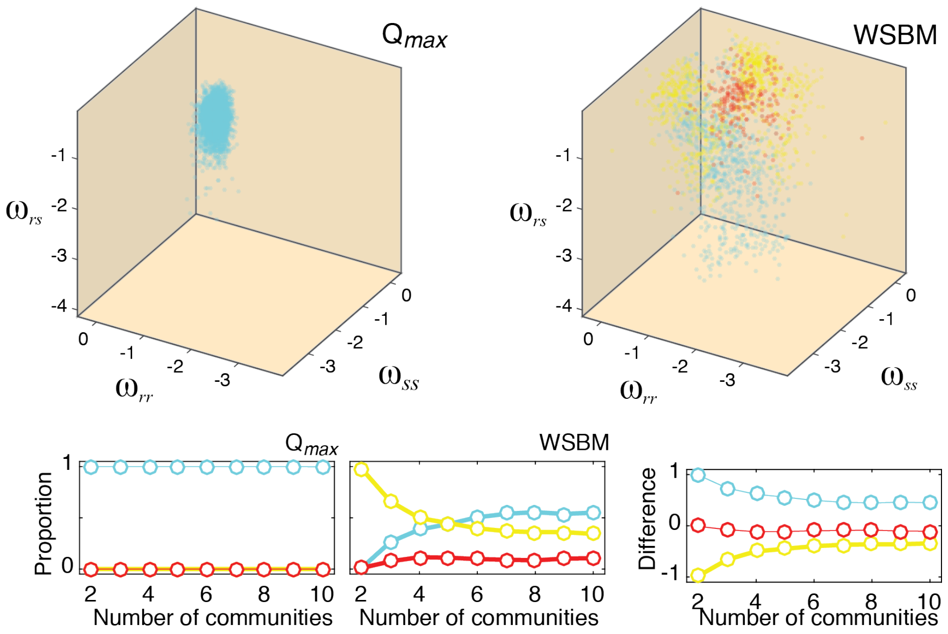

Supplementary Figure 9: **Community morphospace for *C. elegans* connectome.** The top panels depict the community morphospace (in log scale) for community dyads recovered from partitions detected using Q_{max} (left) and the WSBM (right). The color of each point represents each dyad's classification: cyan = assortative, yellow = core-periphery, and red = disassortative. The top plots are depicted with the number of communities fixed at $K = 5$. The bottom panels depict the proportion of dyad classes as we varied the number of communities from $K = 2$ to $K = 10$. In general, Q_{max} only detected assortative community dyads while the WSBM detected all three types.

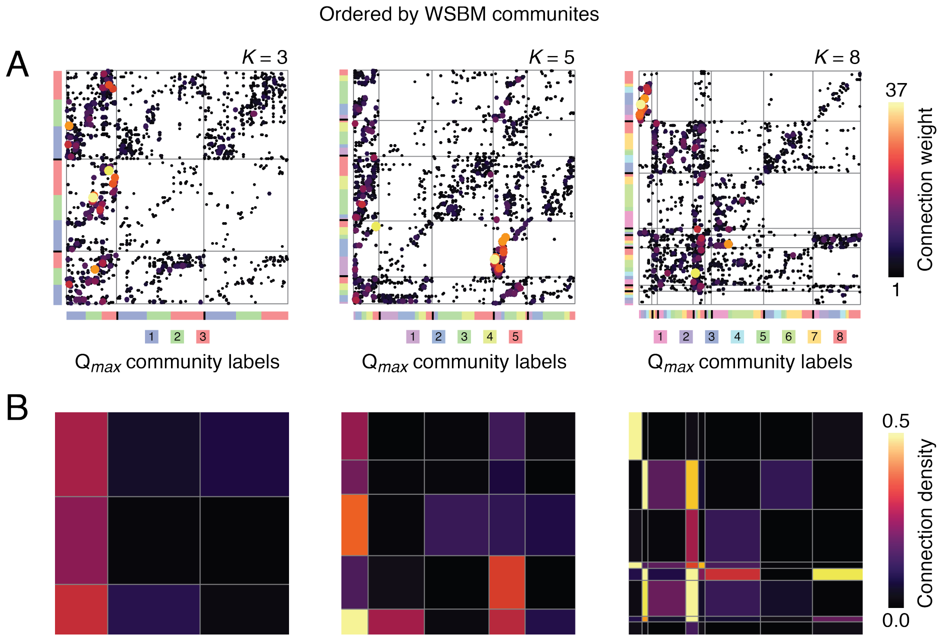

Supplementary Figure 10: ***C. elegans* connectivity matrix reordered by community assignments.** (A) Each panel in the top row depicts the same connectivity matrix of chemical synapses among $N = 279$ neurons of the nematode *C. elegans*. Edge weights represent the number of synapses and are indicated by both the color and size of each edge. The rows and columns of each matrix are ordered according to WSBM community assignments. Along both the x- and y-axes are colored plots. The color of each row and column represents the Q_{max} community assignment of the corresponding neuron. Note: in general, the Q_{max} assignments are heterogeneously distributed across WSBM communities, suggesting an inexact correspondence. (B) Panels in the bottom row depict the connection weight density of the blocks defined by the WSBM community assignments. Note: in general, connection density is not strongest along the diagonal (which would indicate assortative communities). Instead, the density of off-diagonal blocks is sometimes greater than that of the diagonal blocks, which indicates the presence of non-assortative communities.

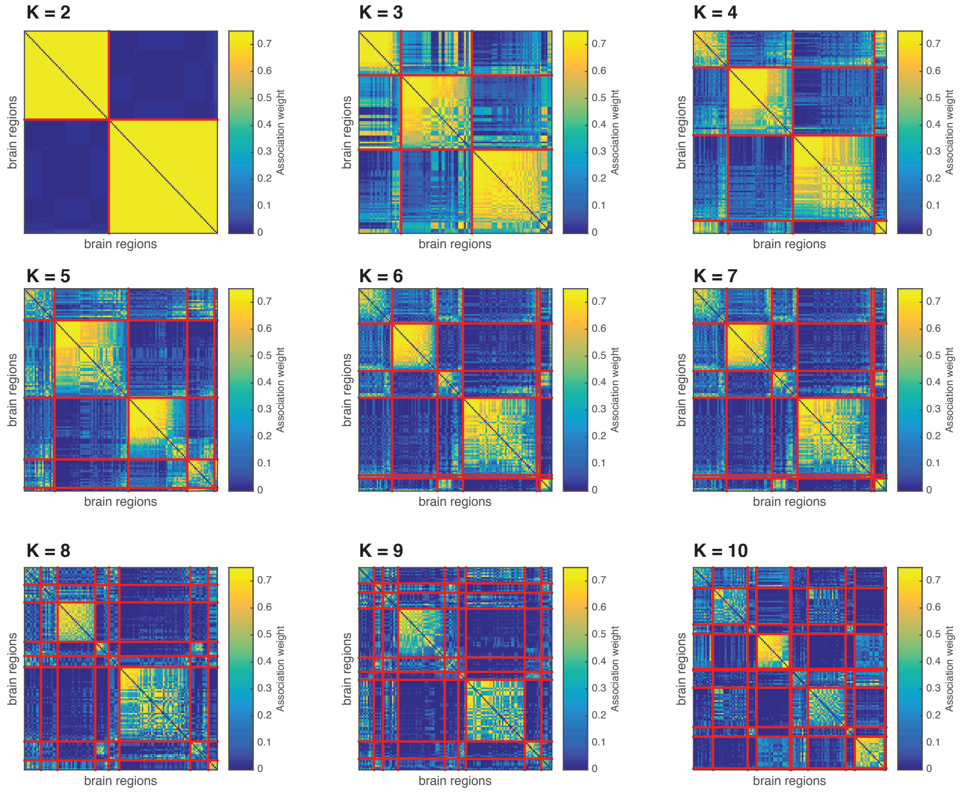

Supplementary Figure 11: **Association matrices computed from partitions output by WSBM.** Each panel depicts a square, brain region $\times$ brain region association matrix, whose elements indicate the fraction of all partitions in which two nodes were co-assigned to the same community. Brain areas are ordered according to a randomly selected partition. Sub-panels correspond to different numbers of communities, $K = 2, \ldots, 10$.

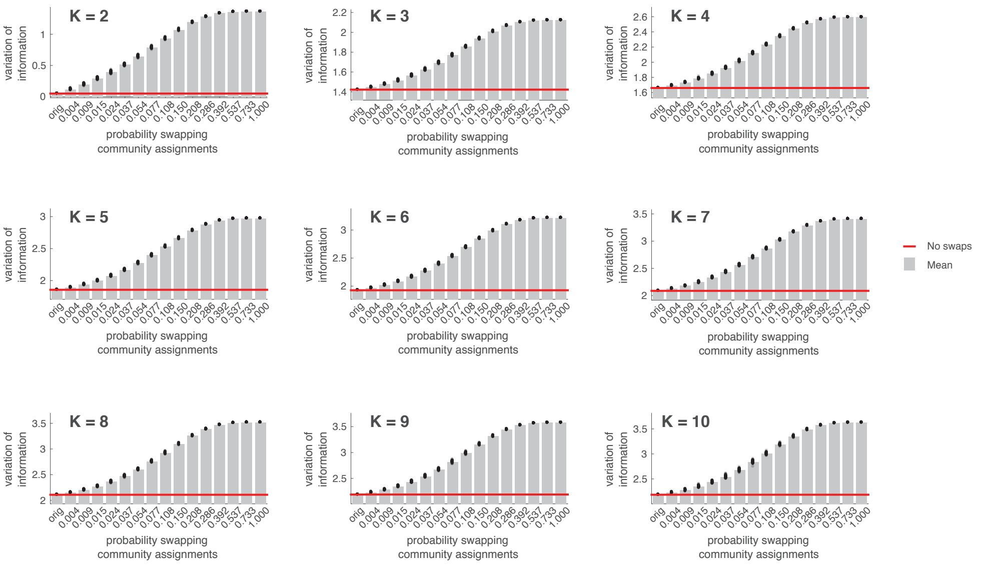

Supplementary Figure 12: **Mean pairwise variation of information (VI) of original and randomized partitions.** For a given number of communities, K, we estimated the mean pairwise VI, which serves as a measure of partition similarity. Lower values of VI imply greater similarity. The VI of the original partitions is shown as a red line. Using a parameterized permutation-based null model, we generate randomized partitions and compute null distributions for the mean pairwise VI. For all values of $K = 2, \ldots, 10$, and for all parameter values, even small changes to community assignments result in statistically significant increases in mean pairwise VI. These observations support the hypothesis that the WSBM algorithm is converging to a set of solutions that are consistent and self-similar.

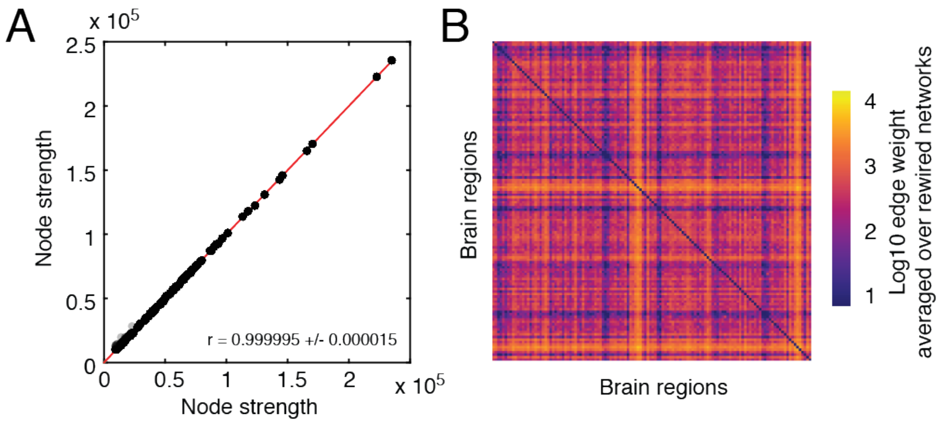

Supplementary Figure 13: **Summary of strength-preserving null models.** (A) Scatterplot showing strong correlation of observed and randomized strength sequences. (B) Mean connectivity matrix obtained by averaging over all 100 realizations of the null model.

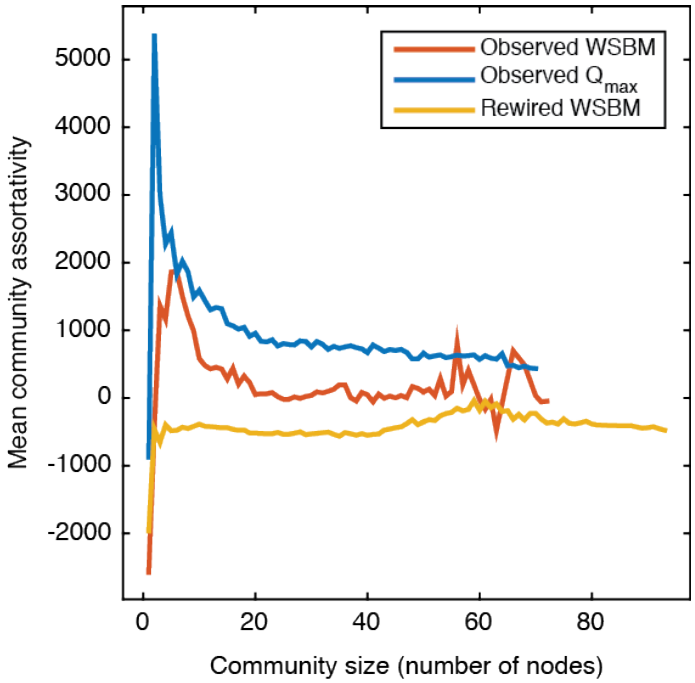

Supplementary Figure 14: **Community assortativity comparisons.** Mean community assortativity curves as a function of community size for Q_{max} and the WSBM applied to the observed network and then for the WSBM applied to the strength-preserved null randomized networks.

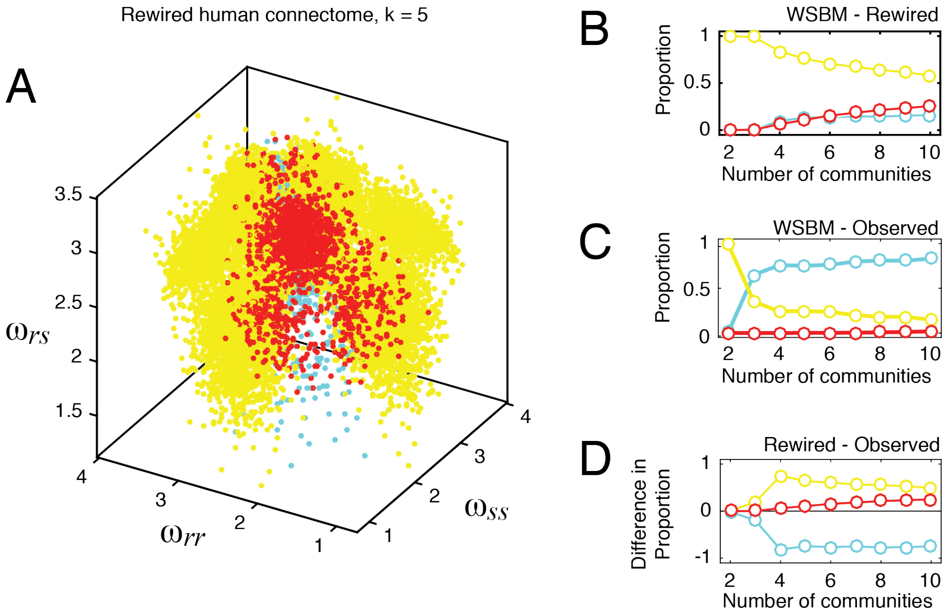

Supplementary Figure 15: **Community morphospace for strength preserved randomized networks.** (A) Each point represents a two-community motif classification. (B) and (C) Proportion of community interaction types at different numbers of communities, $k = 2$ to $k = 10$, for the randomized and observed networks. (D) The difference in community interaction type proportions.

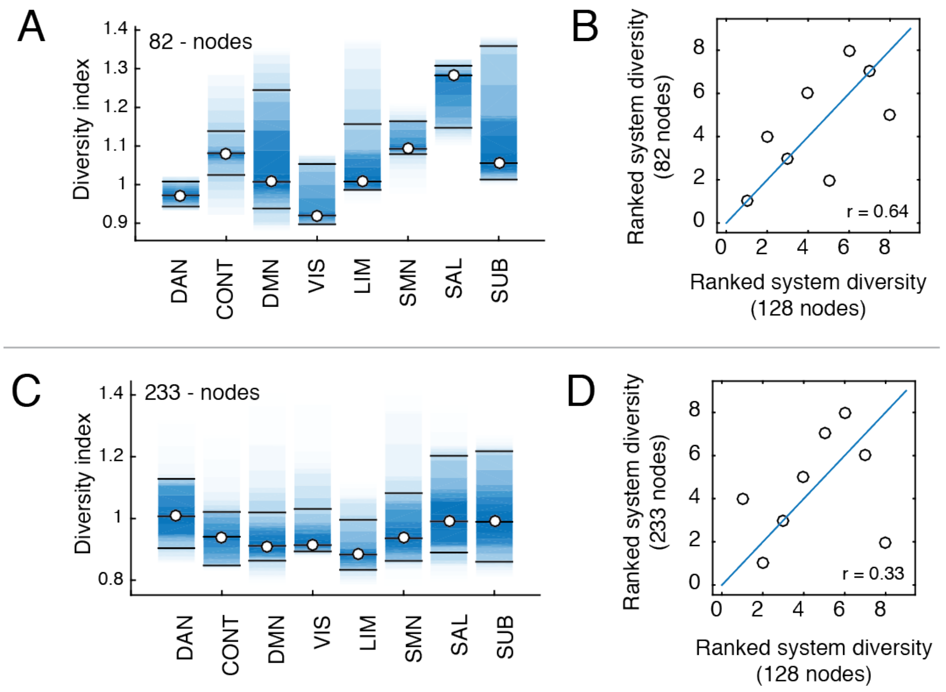

Supplementary Figure 16: **Comparison of system-level diversity scores with 82- and 233-node networks.** (A) System-level diversity scores for 82-node network (B) Rank correlation of system-level scores obtained for the 82-node and 128-node networks. Panels (C) and (D) are the same as (A) and (B) but for the 233-node network.

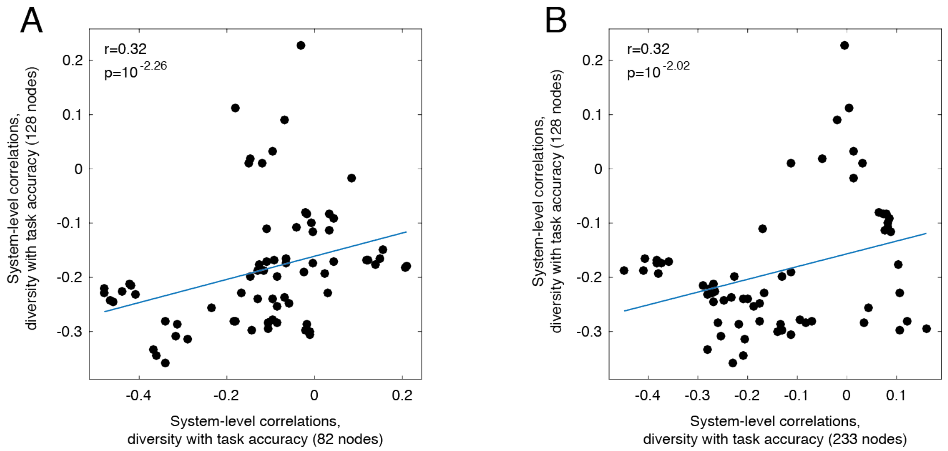

Supplementary Figure 17: **Comparison of system-level correlations of diversity and task performance with 82- and 233-node networks.** (A) Comparison of system-level correlations between the 82- and 128-node networks for all $K = 2$ to $K = 10$. (B) Same as panel (A), but for the 233-node network.

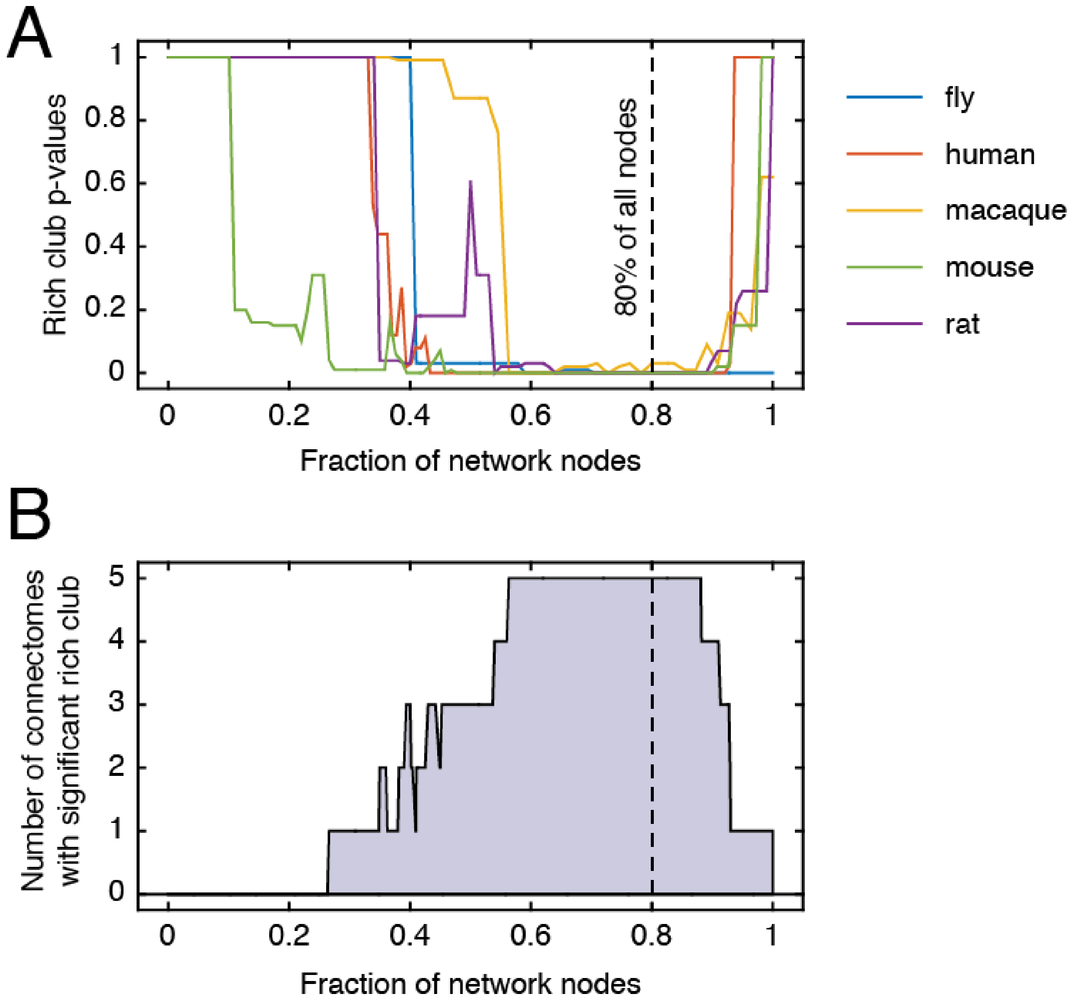

Supplementary Figure 18: **Rich club analysis.** (*A*) P-values for rich clubs across all five species. (*B*) Number of statistically significant rich clubs as a fraction of network size.

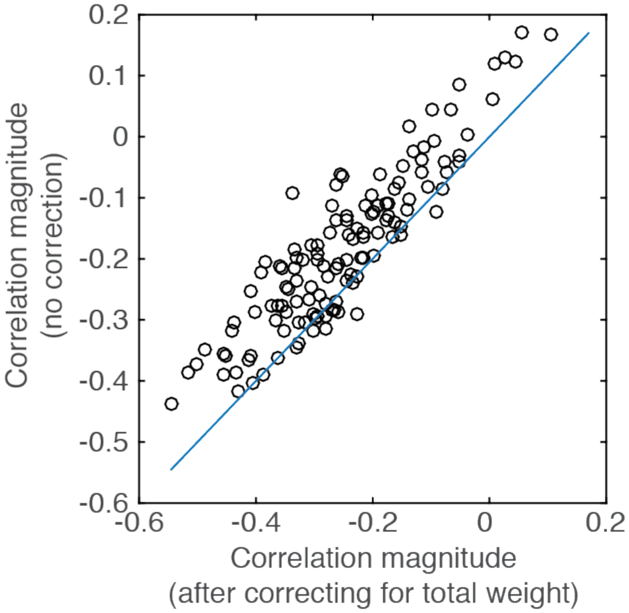

Supplementary Figure 19: **Effect of total network weight corrections on correlations between diversity scores and task performance.** Each point represents the region-level correlation coefficient and the blue line represents the break-even line. If two brain regions had identical correlations with and without corrections for total network weight then they would fall along this line.
